# Eastern Canadian Gastrointestinal Cancer Consensus Conference 2024

**DOI:** 10.3390/curroncol32030175

**Published:** 2025-03-18

**Authors:** Jennifer Leigh, Arwa Ahmed, Francine Aubin, Scott Berry, Melanie Boucher, Marie-Pierre Campeau, Bruce Colwell, Stacie Connors, Jessica Corbett, Shivani Dadwal, Shaan Dudani, Elena Elimova, Conrad Falkson, Luisa Galvis, Rakesh Goel, Joanna Gotfrit, Angela Hyde, Michela Febbraro, David T. Laidley, Gordon Locke, Aamer Mahmud, Thais Baccili Cury Megid, James Michael, Vimoj J. Nair, Stephen Quigley, Ravi Ramjeesingh, Setareh Samimi, Melanie Seal, Stephanie Snow, Silvana Spadafora, Teri Stuckless, Brooke Wilson, Timothy Asmis, Rachel Goodwin, Michael Vickers

**Affiliations:** 1 Mount Sinai Hospital, Toronto, ON M5G 1X5, Canada; 2 The Ottawa Hospital Cancer Centre, Ottawa, ON K1H 8L6, Canada; 3 Centre Hospitalier de l’Universite de Montreal, Montreal, QC H2X 3E4, Canada; 4 Trillium Health Partners, Mississauga, ON L5A 4G1, Canada; 5 Prince Edward Island Cancer Treatment Center, Charlottetown, PE C1A 8T5, Canada; 6 Queen Elizabeth II Health Sciences Center, Halifax, NS B3H 3A7, Canada; 7 Horizon Health Network, Fredericton, NB E3B 4R3, Canada; 8 Juravinski Cancer Center, Hamilton, ON L8V 5C2, Canada; 9 William Osler Health System, Brampton, ON L6R 3J7, Canada; 10 Princess Margaret Cancer Center, Toronto, ON M5G 2M9, Canada; 11 Kingston Health Sciences Center, Kingston, ON K7L 2V7, Canada; 12 Dr. H. Bliss Murphy Cancer Center, St. John’s, NL A1B 3X5, Canada; 13 Algoma District Cancer Program, Sault Ste. Marie, ON P6B 0A8, Canada; 14 London Health Sciences Center, London, ON N6A 5W9, Canada; 15 Dr. Georges-L.-Dumond University Hospital Center, Moncton, NB E1C 2Z3, Canada; 16 Saint John Regional Hospital Oncology Center, Saint John, NB E2L 4L2, Canada; 17 Health Sciences Center-Eastern Health, St. John’s, NL A1B 3V6, Canada; 18 Hopital du Sacre-Coeur de Montreal, Montreal, QC H4J 1C5, Canada

**Keywords:** guidelines, rectal cancer, colorectal cancer, biliary tract cancer, gastroenteropancreatic neuroendocrine tumors, gastroesophageal cancer, chemotherapy, radiation therapy, surgery, HER-2 positive

## Abstract

The Eastern Canadian Gastrointestinal Cancer Consensus Conference was an annual meeting that was held in St. John’s, Newfoundland and Labrador, from 26 to 28 September 2024. This included experts in medical oncology, radiation oncology, surgical oncology, nuclear medicine, and general practitioners in oncology (GPO) from across the eastern Canadian provinces who are involved in the management of patients with gastrointestinal malignancies. This consensus statement generated by the conference addresses multiple topics, including the management of localized rectal cancer, liver-limited colorectal cancer, systemic therapy for advanced biliary tract cancers, radioligand therapy for gastroenteropancreatic neuroendocrine tumors (GEP-NETs), systemic therapy for pancreatic and midgut well-differentiated NETs, and systemic therapy for HER2-positive gastroesophageal cancers.

## 1. Introduction

The annual Eastern Canadian Gastrointestinal Cancer Consensus Conference was held in St. John’s, Newfoundland and Labrador, between 26 and 28 September 2024. The objective of the conference was to develop consensus statements on evolving topics in the management of gastrointestinal and hepatobiliary (HPB) cancers. Conference participants included medical, radiation, and surgical oncologists, nuclear medicine specialists, and general practitioners in oncology (GPOs) from across the eastern Canadian provinces, including Ontario, Quebec, Nova Scotia, Newfoundland and Labrador, New Brunswick, and Prince Edward Island. The conference topics were developed by the organizing committee and speakers prior to the conference and were selected as they represent the most common questions clinicians in eastern Canada face. Each consensus statement was developed following a presentation of the most recent advances in the pertinent literature. Recommendations presented in this paper outline the consensus opinions of the physicians involved in the management of gastrointestinal cancers who participated in this meeting.

For each consensus section, the relevant scientific evidence was presented and discussed amongst meeting participants. Recommendations were formulated as a group and categorized by the level of evidence.

- Level I: Evidence from randomized controlled trials
- Level II-1: Evidence from controlled trials without randomization
- Level II-2: Evidence from analytic cohorts or case–control studies
- Level II-3: Evidence from comparisons between times or places with and without the intervention
- Level III: The opinion of respected authorities based on clinical experience, descriptive

## 2. Multidisciplinary Management of Rectal Cancer (Total Neoadjuvant Therapy/Non-Operative Management (NOM))

Question 1: What are the important considerations involved in deciding a treatment plan for patients with proficient mismatch repair (pMMR), locally advanced rectal cancer?

- We recommend that the management of patients with locally advanced, pMMR rectal cancer be discussed in a multidisciplinary tumor board (MDT) forum that should include colorectal (CRC) surgeons, radiation oncologists, medical oncologists, radiologists, pathologists, and gastroenterologists [Level III]. Important components of an informed patient discussion include the following:
  ○ The patient’s health status, including comorbidities and their performance status
  ○ Patient values, goals, and preferences
  ○ The likelihood of requiring a permanent colostomy or temporary ileostomy
  ○ The chances of the avoidance of total mesorectal excision (TME) surgery with non-operative treatment approaches
  ○ The frequency and types of assessments required for NOM (see details for the assessments in the evidence summary below)
  ○ The risks of local and distant recurrence with NOM

Question 2: What is the optimal sequence of treatment for patients with locally advanced, pMMR rectal cancer pursuing sphincter preservation/NOM?

- If sphincter preservation is the objective, patients with cT3/4, cN+ disease should undergo (chemo)radiotherapy as their initial oncologic intervention, followed by consolidation chemotherapy (fluoropyrimidine- + oxaliplatin-based treatment [1]) [Level I]

### 2.1. Evidence Summary

#### 2.1.1. Non-Operative Management

The definitive treatment for rectal cancer has traditionally involved surgical resection, and for locally advanced disease (T3/T4N0, TxN+), a neoadjuvant treatment approach before radical resection is often utilized to reduce the risk of local recurrence [2]. Non-operative management for locally advanced rectal cancer is a desired treatment approach for patients who wish to increase the likelihood of organ preservation (and for the avoidance of a permanent colostomy) while maintaining their chance of a cure. As shown in the OPRA trial, total neoadjuvant therapy (TNT) using neoadjuvant long-course chemoradiation followed by consolidation chemotherapy (fluoropyrimidine + oxaliplatin) has the best available evidence for organ preservation for appropriate patients [1,3]. The decision for NOM should be a joint informed decision between the patient and their providers, understanding that long-term organ preservation can only be achieved in approximately half of patients with rectal cancer treated with the OPRA protocol [1].

Tumor restaging should occur within 8 weeks (+/−4) of TNT, as per the trial protocol [1]. Patients with a complete or near-complete clinical response to this protocol can safely undergo close surveillance for tumor regrowth/recurrence with a watch-and-wait approach. In this trial, this included flexible sigmoidoscopy every 4 months and pelvic MRI every 4–6 months in the first 2 years, then flexible sigmoidoscopy every 6 months and MRI annually for the following 3 years, as evidence showed that the risk of local recurrence is higher in the first 2 years with this treatment approach [1,3]. A staging CT scan of the chest and abdomen can be performed every 6–12 months in the first 2 years, then annually for the following 3 years. Patients with a residual/incomplete response on initial response assessment, or local recurrence during the watch-and-wait period, should be offered surgery with TME.

#### 2.1.2. Total Neoadjuvant Therapy

Total neoadjuvant therapy is a strategy involving the administration of (chemo)radiation and systemic chemotherapy before surgery. The use of intensified chemotherapy in TNT for locally advanced rectal cancer has also been studied. The PRODIGE-23 phase III trial showed improved disease-free survival (DFS) and overall survival (OS) in locally advanced rectal cancer (cT3/4 cN+) patients when triplet FOLFIRINOX chemotherapy (fluoropyrimidine + oxaliplatin + irinotecan) was used prior to chemoradiation and surgical resection compared to conventional long-course chemoradiation followed by surgical resection [4,5]. The 7-year OS was 81.9% in the neoadjuvant chemotherapy group and 76.1% in the standard of care group (*p* = 0.033) [5]. In addition, there was a higher complete pathological response rate when using TNT compared with chemoradiation (28% vs. 12%, *p* ≤ 0.05). Given the results of the PRODIGE-23 trial, using intensified chemotherapy (FOLFIRINOX) in the TNT approach can be considered in medically fit patients, especially patients with high-risk locally advanced rectal cancer.

Multiple trials have shown the efficacy of long-course chemoradiation therapy as well as short-course radiation therapy in the management of localized rectal cancer. These radiation therapy regimens, however, have not been directly compared in the context of TNT. The RAPIDO trial included patients with rectal adenocarcinoma and high-risk features, which included cT4a/b, cN2, enlarged lateral lymph nodes, extramural vascular invasion, or mesorectal fascia involvement [5]. Patients were randomized to the experimental group, which consisted of short-course radiotherapy followed by chemotherapy (six cycles of CAPOX or nine cycles of FOLFOX), followed by surgery, or to the standard of care arm, which consisted of long-course RT administered concurrently with capecitabine, followed by surgery and then adjuvant chemotherapy as per the institutional policy. After 3 years, there was an improvement in the disease-related treatment failure with short-course radiotherapy (23.7% vs. 30.4%, HR 0.75) [5]. In an update after a 5.6-year median follow-up, the improvement in the disease-related treatment failure driven by a reduction in distant metastatic disease persisted for the TNT arm, but the experimental group was associated with increased local regional relapses (12% vs. 8%, *p* = 0.07) and no improvement in the OS [6,7]. It is important to note that the mesorectum was breached more often in the short-course TNT arm, which may have contributed to this difference. It is not clear if the increase in local or regional control could be improved by the use of long-course chemoradiation therapy because only short-course radiation therapy was used in the TNT arm. We suggest discussing these cases at MCC and also including the patient’s preferences in deciding a TNT regimen.

## 3. Management of Liver-Only Metastatic Colorectal Cancer (mCRC)

Question 1: What are the general surgical principles in the management of metastatic colorectal cancer (mCRC) involving liver-only disease?

- The management of all patients with liver-only mCRC should be discussed in an MDT forum including HPB surgeons, CRC surgeons, radiologists, interventional radiologists, pathologists, gastroenterologists/hepatologists, medical oncologists, and radiation oncologists [Level III]
- Important components of an informed patient discussion include the following [Level III]:
  ○ The patient’s health status, including comorbidities and their performance status
  ○ Synchronous versus metachronous presentation of metastatic disease
  ○ The patient’s biomarker status, including MMR, RAS, and BRAF status
  ○ The patient’s values, goals, and preferences
  ○ The likelihood of cure balanced with the risk of toxicity
  ○ Consideration of resection of the primary could be considered in either a synchronous or staged technique
  ○ How to approach “ghost” (vanishing) lesion(s) if the patient has a good response to neoadjuvant treatment
  ○ The need for neoadjuvant systemic therapy to downstage disease or assess the disease biology

Question 2: What are the liver-directed therapy options that need to be discussed for liver-only mCRC [Level III]?

- Resection
- Ablation
- Resection and ablation
- Ablative radiation (stereotactic body radiation therapy)
- Transplant for unresectable disease
- The consideration of available clinical trials

### Evidence Summary

While the majority of metastatic colorectal cancers are considered incurable, there are a subset of patients with liver-only disease who are potentially curable with surgical resection. This constitutes approximately 20% of patients with liver-limited disease, with 5-year survival rates ranging from 20 to 45% [8,9]. The criteria for resectability depend on a number of technical and oncologic factors, including, but not limited to, whether 30% or more of the liver would be left following R0 resection, the presence of synchronous metastatic sites, the clinical aggressiveness of the tumor, the biomarker status, the patient’s preference, and the patient’s overall health and performance status [8]. A biopsy to confirm that the liver metastases are related to the colorectal primary is not always required if radiographically and clinically the disease is in keeping with mCRC. Given the complexity of these decisions, all cases should be discussed in a multidisciplinary forum that includes surgical, medical, and radiation oncologists, radiologists, interventional radiologists, pathologists, and gastroenterologists/hepatologists. Of note, the best way to manage patients who have received prior NOM for rectal cancer and are found to have liver metastases is unclear, and this specific group of patients was not a specific focus of this consensus.

In patients with favorable oncologic criteria, in whom upfront R0 resection is feasible, the guidelines recommend upfront resection [8]. In those with less favorable disease, the role of neoadjuvant chemotherapy to downstage the tumor or assess the disease biology should be discussed in a multidisciplinary forum. The EORTC Intergroup trial 40983 examined the use of peri-operative FOLFOX in patients with CRC with a primary resected or deemed to be resectable, one to four liver metastases that were potentially resectable, no extrahepatic disease, and no prior oxaliplatin-based chemotherapy. The trial found no significant difference in PFS compared to those who received surgery alone; however, in an exploratory analysis that included only patients who underwent surgery, the HR favored the group who had received chemotherapy (HR 0.73, 95% CI 0.55–0.97) [10]. There was no significant difference in the OS between the groups, and more reversible post-operative complications (e.g., post-operative infection) were seen in the peri-operative chemotherapy group [10]. The New EPOC trial looked at the addition of an EGFR inhibitor (cetuximab) to peri-operative chemotherapy and showed a disadvantage regarding the overall survival with the addition of the EGFR inhibitor [11]. The JCOG0603 trial examined the role of post-operative chemotherapy (FOLFOX) versus surgery alone and again found no significant difference in OS; however, DFS was favored in the chemotherapy arm (HR 0.67, 95% CI 0.50–0.92). Increased adverse events were seen in the chemotherapy arm. Finally, a pooled analysis of two trials using 5-FU following metastectomy compared to resection alone found no DFS or OS difference. However, a multivariate analysis controlling for a number of factors including the number of metastases showed a significant benefit for both OS and DFS compared to post-operative chemotherapy (HR of 0.72) [12]. Factors associated with worsened DFS and OS were an increased number of metastases (two or more) and the treatment group (the surgery-alone group as opposed to the chemotherapy group). The decision to use peri-operative or adjuvant chemotherapy should be discussed with the patient on an individualized basis.

There are a number of liver-directed therapies other than resection that can be discussed with the patient in the appropriate clinical context. These include radiofrequency ablation, stereotactic body radiation therapy (SBRT), transplants for unresectable disease, and the consideration of clinical trials. A systematic review and meta-analysis including eighteen studies that assessed SBRT use in patients with one to five metastatic lesions in the liver and who were not surgical candidates revealed a 2-year OS of 57%, with a local control rate of 60% [13]. However, when only dose-escalated SBRT regimens were analyzed in prospective studies, the 2-year local control rate was 81–100%, respectively [14]. Radiofrequency ablation is another viable option with 5-year survival rates ranging from 14 to 55% [15,16]. The technique does have size limitations, with the recommended maximum lesion being 2–3 cm, and may be ineffective near blood vessels or near the dome of the diaphragm or can be risky when abutting the bowel. This has also been studied retrospectively in combination with resection and has been shown to have a 5-year OS of 37% [17].

Recently, thermal ablation was also compared to surgery in the COLLISION trial, which was a phase III trial randomizing patients with colorectal liver metastasis with 10 or fewer lesions (size of 3 cm or less), no extrahepatic disease, and a good performance status for surgical resection or thermal ablation [18]. This demonstrated no significant differences in OS (HR 1.04) between the two groups, and the number of adverse events, length of hospitalization, and local control all favored the ablation arm (HR 0.18, 95% CI 0.040–0.838, *p* = 0.029). Other local options that have been explored include trans-arterial chemoembolization (TACE) and trans-arterial radioembolization (TARE). The recent EPOCH trial compared second-line chemotherapy to chemotherapy plus TARE in patients with liver metastases and found significantly improved PFS with the combination (HR 0.69, 95% CI 0.54–0.88, *p* = 0.0013) [19]. Hepatic arterial infusion of chemotherapy through intra-arterial pumps has also been explored, with an objective response rate (ORR) of 62% in heavily pre-treated patients [20]. This is not routinely used in Canada except in very specialized centers.

Small trials have also shown favorable OS and DFS outcomes with liver transplantation in highly selective mCRC patients with unresectable liver metastases. The SECA-I study included patients with a complete excision of the primary tumor, ECOG 0–1, and a minimum of 6 weeks of chemotherapy. This study identified risk factors associated with the outcomes of transplantation, which included a carcinoembryonic antigen level of >80 μg/L, progressive disease on chemotherapy, a size of the largest lesion of >5.5 cm, and <2 years having passed from the resection of the primary tumor to transplantation [21]. The SECA-II trial built upon this but narrowed inclusion to those with at least a 10% response to chemotherapy and showed an 83% OS at 5 years [22]. TransMet was a multicenter, prospective, randomized, controlled trial in patients with permanently unresectable colorectal liver metastases from resected non-BRAF-mutated CRC [23]. Patients had to be responsive to 3 or more months of chemotherapy, have had three or fewer lines of therapy, and have no extrahepatic disease. They were randomized to liver transplantation plus chemotherapy or chemotherapy alone. The five-year OS in the intention-to-treat population who received transplantation plus chemotherapy was 56.5% versus 12.6% for the chemotherapy-alone group (HR 0.37). In the per-protocol analysis, the 5-year OS in the combination group was 73.3% versus 9.3% in the chemotherapy-alone group. Therefore, liver transplantation is another option for selected patients (who would meet trial eligibility), and referral for transplant consideration should be performed early [21,22,24].

## 4. Systemic Therapy of Biliary Tract Cancers

Question 1: What are the current first-line considerations and management options in advanced biliary tract cancers?

- The optimal first-line treatment for patients with no immunotherapy contraindications includes cisplatin and gemcitabine chemotherapy with immunotherapy (durvalumab or pembrolizumab) [25,26] [Level I]
- If a patient has a contraindication to immunotherapy, then cisplatin and gemcitabine chemotherapy alone can be utilized. If there is a contraindication to cisplatin, other platinum-based regimens can be considered [27,28] [Level I]
- Biomarker testing (including NGS and IHC for HER-2 and MMR status) should be completed as part of the diagnostic work-up to identify patients eligible for targeted therapy clinical trials and to inform second-line options [Level I].
- We endorse that biomarker testing should be publicly funded and available for all patients [Level III]
- Clinical trials may be an option and should be discussed with patients
- Best supportive care is also an option and should be discussed with patients

Question 2: What is the role of biomarker profiling in advanced biliary tract cancers?

- The role of biomarker profiling is to identify therapeutic options, including clinical trials and accessible targeted treatments
- Patients who harbor an IDH1 mutation should be treated with an IDH1 inhibitor (Ivosidenib) based on randomized phase III evidence [29] [Level I]
- Based on single-arm phase II data, following first-line treatment, the following agents could be considered for the appropriate mutation/alteration [Level II-1]
  ○ An FGFR2 inhibitor (e.g., Pemigatinib) [30]
  ○ An NTRK inhibitor (e.g., Larotrectinib, Entrectinib) [31,32]
  ○ HER-2-targeted therapy (e.g., trastuzumab–deruxtecan, pertuzumab + trastuzumab, tucatinib) [33,34,35]
- Patients who have deficient MMR disease and have not been exposed to immunotherapy in the first-line setting should be considered for treatment with immunotherapy in the second-line setting [36] [Level II-1]
- Patients who harbor other actionable mutations (BRAF, etc.) should be considered for targeted therapy through clinical trials, compassionate access, or other means of access [Level III]

Question 3: What is the recommendation for second-line treatment for advanced BTC for patients with no actionable genomic alteration identified?

- If no actionable genomic alteration is identified, then FOLFOX should be considered as a treatment option after progression on gemcitabine–cisplatin-based therapy [37] [Level II-1]
- Another potential option based on a randomized phase IIB trial also demonstrating an overall survival benefit is NALIRI + 5FU [38] [Level II-1]
- Available clinical trials are an option and should be discussed with patients
- Best supportive care is an option and should be discussed with patients

### Evidence Summary

Primary biliary tract cancers are rare and account for less than 1% of cancers worldwide [39] (p. 20). In the first-line metastatic setting, the standard of care treatment is a combination of cisplatin, gemcitabine, and immunotherapy, or cisplatin and gemcitabine alone in those with contraindications to immunotherapy. In both the UK ABC-02 and Japanese BT22 studies, the cisplatin–gemcitabine combination was shown to provide an OS benefit over gemcitabine monotherapy [27,28]. The TOPAZ-1 trial then built upon this by randomizing patients to cisplatin–gemcitabine with or without durvalumab. This demonstrated an improved mOS from 11.3 months to 12.9 months (HR 0.76) [25]. Keynote-966 evaluated cisplatin–gemcitabine with or without pembrolizumab and found that the addition of immunotherapy improved the mOS from 10.9 months to 12.7 months (HR 0.83) [26] (p. 966). In patients with cisplatin contraindications, other platinum-based regimens can be considered as there are data supporting their efficacy [40]. The alternative options of either clinical trials or best supportive care should also be discussed with patients. Additionally, baseline biomarker testing (NGS including validated testing for FGFR fusions, IHC for HER-2 and dMMR) should be funded and available to all patients up front to inform their clinical trial options, as well as to anticipate treatment options in the second-line setting.

In the second-line setting, treatment options are largely guided by the biomarker status. Approximately 40% of patients will harbor an actionable mutation. Mutations in IDH1 or IDH2 account for approximately 10–20% of these targetable mutations. The oral IDH1 inhibitor Ivosidenib was studied in the phase III ClarIDHy trial, which showed significantly improved progression-free survival (PFS) (2.7 vs. 1.4 months, HR 0.37), with no statistically significant OS benefit in the overall cohort [29]. When adjusted for crossover, however, an OS benefit was seen (mOS 10.3 vs. 5.1 months, HR 0.49). This drug is not currently available in Canada. There are phase II data supporting the use of other targeted agents, specifically Pemigatinib for FGFR2 fusions, Larotrectinib or Entrectinib for NTRK fusions, and trastuzumab–deruxtecan for HER-2-amplified disease [30,31,32,34]. Each of these could be considered for patients with the applicable mutation. Ongoing phase III trials with these targeted agents will provide more definitive evidence. Lastly, the Keynote 158 trial examined the use of pembrolizumab in MSI-H patients with non-colorectal cancers who progressed on prior therapy, including biliary tract cancers, and demonstrated a benefit, with an ORR of 40.9% and an mOS of 24.3 months [41] (p. 15). This should be considered for those who did not receive immunotherapy in the first-line setting. Patients who harbor other actionable mutations such as BRAF should be considered for targeted treatment through clinical trials or compassionate access.

For patients with no actionable mutation, FOLFOX could be considered as an option after progression on cisplatin–gemcitabine. A survival benefit was seen for this regimen versus active symptom management in the ABC-06 phase III trial, although this benefit was modest (mOS 6.2 months versus 5.3 months, HR 0.69) [37]. NALIRI-5-FU could also be considered as an option. The phase IIb NIFTY trial demonstrated that NALIRI-5-FU resulted in improved PFS (4.2 versus 1.7 months, HR 0.56) and OS (8.6 versus 5.3 months, HR 0.68) compared to 5-FU alone [42]. Importantly, the phase II trial NALIRICC also compared NALIRI-5-FU to 5-FU alone and found that it did not improve the PFS or OS [38]. Notably, not all of the options discussed for after first-line therapy are available in all jurisdictions across Canada. Finally, both clinical trials and best supportive care are always options that can be discussed.

## 5. Radioligand Therapy (RLT)/Peptide Receptor Radionuclide Therapy (PRRT) Treatment for Gastroenteropancreatic Neuroendocrine Tumors (GEP-NETs)

Question 1: What is the role of PRRT in well-differentiated metastatic GEP-NETs?

- Decisions regarding PRRT should be integrated into a comprehensive, multidisciplinary treatment plan involving medical oncologists, nuclear medicine, radiation oncology, endocrinologists, oncological surgeons, and pathologists to ensure optimal patient outcomes [Level III]
- RLT/PRRT is an effective treatment option for patients with advanced, well-differentiated neuroendocrine tumors (NETs) expressing somatostatin receptors (SSTRs)
- We endorse the use of PRRT in the following settings:
  ○ Well-differentiated SSTR-positive GEP-NETs after progression on somatostatin receptor analogs [43] (p. 1) [Level I]
  ○ A first-line treatment option for patients with well-differentiated grade 2 and 3 SSTR-positive GEP-NETs considering the disease burden and the disease-related symptoms [44] (p. 2) [Level I]
- Eligible patients for PRRT treatment should receive four cycles of PRRT (Lu-DOTATATE) administered every 8 weeks. They should have adequate renal function (creatinine clearance above or equal to 50 mL/min), hepatic function, and bone marrow reserves [43] (p. 1) [Level I]

### Evidence Summary

PRRT treatment with ^177^Lu-DOTATATE (^177^Lutetium Dotatate) is an approved treatment for patients with advanced well-differentiated midgut NET after progression on SSAs. The efficacy of PRRT was proven by the results of the NETTER-1 phase III study, which showed improved PFS and a higher response rate with ^177^Lu-DOTATATE treatment plus the standard dose of Octreotide compared to a high dose of Octreotide alone [45]. The reported PFS at month 20 was 65.2% favoring the ^177^Lu-DOTATATE treatment arm compared with 10.8% for the high-dose Octreotide arm. In a longer-term follow-up, the median OS was not statistically significant in the final analysis of NETTER-1, with a median OS of 48 months vs. 36.3 months in favor of the Lu-DOTATATE treatment arm. Although not statistically significant, this could be considered clinically important for symptomatic patients with a high disease burden [43]. Additionally, there was 36% crossover from the standard therapy to the PRRT arm, which may also have impacted these results. PRRT is emerging as a first-line treatment for patients with grade 2 and 3 well-differentiated GEP-NETs, as shown by the phase 3 randomized NETTER-2 study [44]. NETTER-2 showed a significant improvement in PFS with ^177^Lu-DOTATATE therapy when added to standard-dose Octreotide compared to high-dose Octreotide alone, with a median PFS of 22.8 months compared to 8.5 months.

PRRT maintenance treatment with ^177^Lu-DOTATATE after the conventional induction four-cycle treatment is still under study. A phase II study published in 2021 conducted by a group in London, Ontario, showed a longer median PFS of 36.1 months with a maintenance low dose of Lu-DOTATATE after the induction treatment [46]. Although the study included patients with well-differentiated NETs from different primary sites, not only GI ones, the median PFS for the midgut NET was 47.7 months, which is higher than the reported PFS of 28.4 months in the NETTER-1 study, and the median PFS for the pancreatic NET was 36.5 months. Despite the encouraging results from this study, the small number of patients and the different primary tumor sites included limit definitive recommendations for maintenance therapy with ^177^Lu-DOTATATE at this time.

PRRT is generally well tolerated, with potential side effects including fatigue, bone marrow suppression, the transient elevation of the liver enzymes, and renal toxicity, especially in patients who have baseline impaired renal function. In the NETTER-1 study, the reported incidences of grade 3 and 4 hematological toxicities with ^177^Lu-DOTATATE treatment were low, with neutropenia rates of 1%, thrombocytopenia rates of 2%, and lymphopenia rates of 9%. Patients receiving PRRT require a long-term follow-up, including periodic imaging and laboratory assessments, to monitor their treatment response, detect disease progression, and manage late-onset side effects.

The sequencing of PRRT and liver-directed therapy in well-differentiated NETs has not been clearly assessed in clinical trials to date. These decisions often depend on the extent of the disease, baseline organ function, availability of treatment modalities, and patient preferences. Liver-directed therapy with locoregional treatment using embolization or ablation can be considered in patients with liver-only metastatic disease or patients with limited extrahepatic metastasis [47,48,49,50,51]. This approach can improve the disease control rate, though the impact on the overall survival is still unknown.

## 6. Systemic Therapy for Pancreatic and Midgut Well-Differentiated NET Treatment

Question 1: What are the systemic therapy options for advanced pancreatic well-differentiated neuroendocrine tumors (pNETs)?

- The management of pNETs should be discussed in a multidisciplinary forum that includes medical oncologists, hepatobiliary surgeons, radiation oncologists, pathologists, nuclear medicine specialists, and radiologists (including interventional radiologists) to decide the optimal management strategy
- The treatment options for unresectable, locally advanced or metastatic well-differentiated pNETs are the following:
  ○ Somatostatin analogs (SSAs): The standard first-line treatment for patients with well-differentiated pNETs [52] [Level I]
  ○ RLT/PRRT: A treatment option for patients with SSTR-positive disease; it can be used after progression on SSAs or as a first-line treatment in grade 2 or 3 (ki67 > 10%) disease [43] [Level I]
  ○ Chemotherapy: Chemotherapy with capecitabine plus temozolomide can be used upon progression on SSAs or as a first-line treatment in patients with aggressive disease when a more rapid clinical response is required [53] [Level I]
  ○ Targeted therapy: The available options are Everolimus, Sunitinib, or Cabozantinib, and they can be used after disease progression on first-line treatment [54,55] [Level I]

Question 2: What are the systemic therapy options for advanced, well-differentiated midgut neuroendocrine tumors (mNETs)?

- The management of mNETs should be discussed in a multidisciplinary forum that includes medical oncologists, oncological surgeons, radiation oncologists, pathologists, nuclear medicine specialists, and radiologists to decide the optimal management strategy
- The treatment options for unresectable locally advanced or metastatic well-differentiated mNETs are the following:
  ○ Somatostatin analogs (SSAs): The standard first-line treatment for patients with well-differentiated mNETs [52,56] [Level I]
  ○ Targeted therapy: The available options for progressing disease on SSAs are Everolimus [Level I], Cabozantinib [Level I], or Lenvatinib [Level II] [54,55,57]
  ○ RLT/PRRT: A treatment option for patients with SSTR-positive disease; it can be used after progression on SSA or as a first-line treatment in grade 2 or 3 disease [43,44] [Level I]
  ○ Chemotherapy: There is insufficient evidence for the routine use of chemotherapy, but it could be considered in the appropriate clinical scenario.

### Evidence Summary

The management of well-differentiated GEP-NETs can be challenging. Clinical cases should be managed in a multidisciplinary fashion, and cases should be presented to a multidisciplinary tumor board with expertise on the management of NET patients. Observation and watch-and-wait approaches might be appropriate for patients with asymptomatic disease progression and good clinical condition. Although well-differentiated NETs are a slow-growing disease, changes in tumor differentiation or proliferation may occur during the course of the disease. Repeating a biopsy or repeating functional images should be considered when clinically indicated. Liver-directed therapy with locoregional treatment using embolization, ablation, or radiotherapy can be considered in patients with liver-only metastatic disease or patients with limited extrahepatic metastasis.

Systemic treatment for advanced cases should be offered to patients when surgery is not feasible. Somatostatin analogs (SSAs) such as Octreotide or Lanreotide are considered to be the standard first-line treatments for well-differentiated pancreatic and midgut NETs. In the PROMID study, Octreotide improved disease control in metastatic well-differentiated midgut NETs, and Lanreotide, as seen in the CLARINET study, improved the PFS compared to a placebo with a PFS of 32.8 months vs. 18 months in pancreatic and intestinal well-differentiated NETs [52,56,58].

After first-line treatment with SSAs, there are a number of available treatment options, albeit with a lack of direct comparison data. Decisions regarding treatment beyond progression on SSAs depend on many factors, including the extent of the disease, disease-related symptoms, treatment availability, patient preferences, and the clinical condition of the patient/patient’s comorbidities. Other important factors include whether or not the disease is SSTR-positive and whether the disease is considered functional or non-functional. RLT/PRRT is an effective treatment option in advanced well-differentiated midgut NETs after progression on SSAs. All patients included in the NETTER-1 study had SSTR-positive disease on baseline In-11 Octreotide or Ga-68 DOTATATE imaging [45]. Chemotherapy has also been studied in the treatment of advanced well-differentiated GEP-NETs. In pNETs in particular, the combination of capecitabine plus temozolomide (CAPETEM) showed an improvement in PFS when compared to temozolomide alone, 22.7 months vs. 14.4 months, with a good tolerance and safety profile [53].

Both RLT/PRRT and the CAPETEM chemotherapy regimen are effective second-line treatment options after progression on an SSA. For pNETs, there are data suggesting that grade 3, well-differentiated pNETs may have a better response to CAPETEM chemotherapy compared to RLT/PRRT [59]. There is a lack of evidence supporting the use of chemotherapy in advanced, well-differentiated midgut NETs; however, it is commonly used, and is recommended in some international guidelines [60].

Several oral systemic therapies have been studied in the treatment of NETs. Everolimus was the first targeted treatment studied in the RADIANT-4 phase III study showing an improvement in PFS in advanced, well-differentiated gastrointestinal NETs progressing on SSAs [54]. Recently, Cabozantinib has emerged as an effective targeted treatment for well-differentiated NETs in the second-line setting and beyond. The Cabinet study showed that Cabozantinib was associated with an impressive PFS improvement in patients with pNETs, with a median PFS of 13.8 months (compared with 4.4 months with a placebo) and 8.4 months in extra-pancreatic NETs (compared with 3.9 months with a placebo) [55]. The use of Lenvatinib was also studied in the phase II TALENT trial, which demonstrated an ORR of 29.9% and an mPFS of 15.7 months in patients with grade I or II GEP-NETs [57].

The treatment of grade 3, well-differentiated GEP NETs is challenging since SSAs may not be as effective as in grade 1 or 2 disease. NETTER-2 looked at a subset of patients with grade 2 and 3 GEP-NETs and found a significant PFS improvement using first-line Lu-DOTATATE therapy in combination with Octreotide compared to high-dose Octreotide alone [44]. Chemotherapy with CAPETEM is also a treatment option for more aggressive disease or grade 3 pNETs.

Later lines of therapy are commonly offered to patients with NETs. However, the use of immunotherapy for the treatment of well-differentiated GEP NETs is not recommended outside clinical trials, as it has not to date been shown to be effective [61]. The role of continuing or discontinuing SSAs as a maintenance therapy for non-functional progressing patients is unknown and is now being tested in a clinical trial (STOPNET).

## 7. Management of HER2+ve Gastroesophageal Cancers

Question 1: What is the recommended first-line management for patients with HER2+ve gastroesophageal cancers?

- All patients with gastric or GEJ cancer must have the following biomarkers tested reflexively and synchronously [Level I]:
  ○ HER-2
  ○ MMR
  ○ PDL1
- Pembrolizumab, in addition to platinum-based chemotherapy and trastuzumab, is recommended for patients with locally advanced unresectable or metastatic gastric or GEJ adenocarcinoma with a CPS greater than or equal to one (without contraindications to immunotherapy) [62] [Level I]
- Patients who are fit with metastatic or unresectable disease should be considered for enrollment onto clinical trials [Level III]
- We do not endorse the use of HER2-directed therapy in the neoadjuvant or adjuvant setting, except in the context of clinical trials

### Evidence Summary

The management of advanced gastroesophageal cancers has become increasingly biomarker-driven and upfront information on a patients’ biomarker status is critical to decision making. HER2 overexpression will be identified in about 20% of gastroesophageal cancers [63]. HER2 positivity is defined as immunohistochemistry (IHC) 3+, IHC2+/fluorescence in situ hybridization-positive, or IHC 2+/silver in situ hybridization-positive. A positive HER2 result has critical treatment implications and thus should be tested upfront. Other important biomarkers include the programmed death ligand 1 (PD-L1) CPS and mismatch repair status (MMR), both of which also have critical treatment implications. Finally, CLDN18.2 is the newest biomarker with targeted treatment options available (zolbetuximab) and is currently undergoing the approval process in Canada [64].

For patients with advanced HER2+ gastroesophageal cancers, it is recommended that they receive HER-2-directed therapy in the first-line setting [65]. This was initially based on the ToGA (trastuzumab for gastric cancer) trial, which was a phase III, multicenter trial including patients with HER2+ gastroesophageal cancer who were randomized to platinum and 5-FU doublet chemotherapy, with or without the addition of trastuzumab, for six cycles, followed by maintenance with trastuzumab for those randomized to that group [63]. The addition of trastuzumab increased the mOS from 11.1 months to 13.8 months (HR 0.74). In a pre-planned exploratory analysis, patients with high HER-2 expression had a longer OS than patients with low HER-2 expression. More recently, the phase III clinical trial KEYNOTE-811 demonstrated a further survival benefit with the addition of pembrolizumab to standard systemic therapy in HER2+ disease (fluoropyrimidine, platinum, and trastuzumab) [62] (p. 8). The final analysis demonstrated an mOS of 20.0 months versus 16.8 months, with an HR of 0.80, in the overall population. In patients with a CPS of 1 or greater, the mOS was 20.1 versus 15.7 months (HR 0.79). The degree of the benefit from the addition of immunotherapy based on the degree of the PD-L1 status in the CPS-positive patients is not clear and should be further studied. In patients with a CPS of less than 1, there was no difference in the mOS between the groups (9.5 months versus 9.5 months, HR 1.03).

In the curative intent setting, the addition of HER2-targeted therapies is not currently the standard of care as there have been no clear DFS or OS benefits to date. The phase III RTOG 1010 trial randomized patients with HER2+ esophageal adenocarcinoma to the CROSS regimen with or without the addition of trastuzumab and found no significant disease-free survival (DFS) benefit [66]. There have been a number of phase II trials that have looked at a dual HER2 blockade in the peri-operative setting with trastuzumab and pertuzumab (PETRARCA, INNOVATION). Both trials did demonstrate an increase in the rates of a pathologic complete response (pCR), although only with single-agent trastuzumab in INNOVATION [67,68]. The toxicity profiles in both studies, however, were unfavorable, and thus anti-HER2 therapy in the peri-operative setting is not recommended outside of a clinical trial.

## References

[1] Verheij F.S., Omer D.M., Williams H., Lin S.T., Qin L.-X., Buckley J.T., Thompson H.M., Yuval J.B., Kim J.K., Dunne R.F. (2024). Long-Term Results of Organ Preservation in Patients With Rectal Adenocarcinoma Treated With Total Neoadjuvant Therapy: The Randomized Phase II OPRA Trial. J. Clin. Oncol..

[2] Sauer R., Becker H., Hohenberger W., Rodel C., Wittekind C., Fietkau R., Martus P., Tschmelitsch J., Hager E., Hess C.F. (2004). Preoperative versus Postoperative Chemoradiotherapy for Rectal Cancer. N. Engl. J. Med..

[3] Garcia-Aguilar J., Patil S., Gollub M.J., Kim J.K., Yuval J.B., Thompson H.M., Verheij F.S., Omer D.M., Lee M., Dunne R.F. (2022). Organ Preservation in Patients With Rectal Adenocarcinoma Treated With Total Neoadjuvant Therapy. J. Clin. Oncol..

[4] Conroy T., Bosset J.F., Etienne P.L., Rio E., François E., Mesgouez-Nebout N., Vendrely V., Artignan X., Bouché O., Gargot D. (2021). Neoadjuvant chemotherapy with FOLFIRINOX and preoperative chemoradiotherapy for patients with locally advanced rectal cancer (UNICANCER-PRODIGE 23): A multicentre, randomised, open-label, phase 3 trial. Lancet Oncol..

[5] Conroy T., Castan F., Etienne P.L., Rio E., Mesgouez-Nebout N., Evesque L., Vendrely V., Artignan X., Bouché O., Gargot D. (2024). Total neoadjuvant therapy with mFOLFIRINOX versus preoperative chemoradiotherapy in patients with locally advanced rectal cancer: Long-term results of the UNICANCER-PRODIGE 23 trial. Ann. Oncol..

[6] Bahadoer R.R., Dijkstra E.A., Van Etten B., Marijnen C.A.M., Putter H., Kranenbarg E.M.-K., Roodvoets A.G.H., Nagtegaal I.D., Beets-Tan R.G.H., Blomqvist L.K. (2021). Short-course radiotherapy followed by chemotherapy before total mesorectal excision (TME) versus preoperative chemoradiotherapy, TME, and optional adjuvant chemotherapy in locally advanced rectal cancer (RAPIDO): A randomised, open-label, phase 3 trial. Lancet Oncol..

[7] Dijkstra E.A., Nilsson P.J., Hospers G.A.P., Bahadoer R.R., Kranenbarg E.M.-K., Roodvoets A.G., Putter H., Berglund Å., Cervantes A., Crolla R.M. (2023). Locoregional Failure During and After Short-course Radiotherapy Followed by Chemotherapy and Surgery Compared With Long-course Chemoradiotherapy and Surgery: A 5-Year Follow-up of the RAPIDO Trial. Ann. Surg..

[8] Cervantes A., Adam R., Roselló S., Arnold D., Normanno N., Taïeb J., Seligmann J., De Baere T., Osterlund P., Yoshino T. (2023). Metastatic colorectal cancer: ESMO Clinical Practice Guideline for diagnosis, treatment and follow-up. Ann. Oncol..

[9] Kanas G.P., Taylor A., Primrose J.N., Langeberg W., Kelsh M., Mowat F., Alexander D., Choti M., Poston G. (2012). Survival after liver resection in metastatic colorectal cancer: Review and meta-analysis of prognostic factors. Clin. Epidemiol..

[10] Nordlinger B., Sorbye H., Glimelius B., Poston G.J., Schlag P.M., Rougier P., Bechstein W.O., Primrose J.N., Walpole E.T., Finch-Jones M. (2008). Perioperative chemotherapy with FOLFOX4 and surgery versus surgery alone for resectable liver metastases from colorectal cancer (EORTC Intergroup trial 40983): A randomised controlled trial. Lancet.

[11] Bridgewater J.A., Pugh S.A., Maishman T., Eminton Z., Mellor J., Whitehead A., Stanton L., Radford M., Corkhill A., Griffiths G.O. (2020). Systemic chemotherapy with or without cetuximab in patients with resectable colorectal liver metastasis (New EPOC): Long-term results of a multicentre, randomised, controlled, phase 3 trial. Lancet Oncol..

[12] Mitry E., Fields A.L.A., Bleiberg H., Labianca R., Portier G., Tu D., Nitti D., Torri V., Elias D., O’Callaghan C. (2008). Adjuvant Chemotherapy After Potentially Curative Resection of Metastases From Colorectal Cancer: A Pooled Analysis of Two Randomized Trials. J. Clin. Oncol..

[13] Petrelli F., Comito T., Barni S., Pancera G., Scorsetti M., Ghidini A. (2018). Stereotactic body radiotherapy for colorectal cancer liver metastases: A systematic review. Radiother. Oncol..

[14] Vera R., González-Flores E., Rubio C., Urbano J., Camps M.V., Ciampi-Dopazo J.J., Rincón J.O., Macías V.M., Braco M.A.G., Suarez-Artacho G. (2020). Multidisciplinary management of liver metastases in patients with colorectal cancer: A consensus of SEOM, AEC, SEOR, SERVEI, and SEMNIM. Clin. Transl. Oncol..

[15] Shady W., Petre E.N., Do K.G., Gonen M., Yarmohammadi H., Brown K.T., Kemeny N.E., D’Angelica M., Kingham P.T., Solomon S.B. (2018). Percutaneous Microwave versus Radiofrequency Ablation of Colorectal Liver Metastases: Ablation with Clear Margins (A0) Provides the Best Local Tumor Control. J. Vasc. Interv. Radiol..

[16] Wong S.L., Mangu P.B., Choti M.A., Crocenzi T.S., Dodd G.D., Dorfman G.S., Eng C., Fong Y., Giusti A.F., Lu D. (2010). American Society of Clinical Oncology 2009 Clinical Evidence Review on Radiofrequency Ablation of Hepatic Metastases From Colorectal Cancer. J. Clin. Oncol..

[17] Evrard S., Poston G., Kissmeyer-Nielsen P., Diallo A., Desolneux G., Brouste V., Lalet C., Mortensen F., Stättner S., Fenwick S. (2014). Combined Ablation and Resection (CARe) as an Effective Parenchymal Sparing Treatment for Extensive Colorectal Liver Metastases. PLoS ONE.

[18] Meijerink M.R., Van Der Lei S., Dijkstra M., Versteeg K.S., Buffart T.E., Lissenberg-Witte B.I., Swijnenburg R.-J., Tol M.P.v.D., Puijk R.S. (2024). Surgery versus thermal ablation for small-size colorectal liver metastases (COLLISION): An international, multicenter, phase III randomized controlled trial. J. Clin. Oncol..

[19] Mulcahy M.F., Mahvash A., Pracht M., Montazeri A.H., Bandula S., Martin R.C.G., Herrmann K., Brown E., Zuckerman D., Wilson G. (2021). Radioembolization With Chemotherapy for Colorectal Liver Metastases: A Randomized, Open-Label, International, Multicenter, Phase III Trial. J. Clin. Oncol..

[20] Boige V., Malka D., Elias D., Castaing M., De Baere T., Goere D., Dromain C., Pocard M., Ducreux M. (2008). Hepatic Arterial Infusion of Oxaliplatin and Intravenous LV5FU2 in Unresectable Liver Metastases from Colorectal Cancer after Systemic Chemotherapy Failure. Ann. Surg. Oncol..

[21] Dueland S., Guren T.K., Hagness M., Glimelius B., Line P.-D., Pfeiffer P., Foss A., Tveit K.M. (2015). Chemotherapy or liver transplantation for nonresectable liver metastases from colorectal cancer?. Ann. Surg..

[22] Dueland S., Syversveen T., Solheim J.M., Solberg S., Grut H., Bjørnbeth B.A., Hagness M., Line P.-D. (2020). Survival Following Liver Transplantation for Patients With Nonresectable Liver-only Colorectal Metastases. Ann. Surg..

[23] Adam R., Piedvache C., Chiche L., Adam J.P., Salamé E., Bucur P., Cherqui D., Scatton O., Granger V., Ducreux M. (2024). Liver transplantation plus chemotherapy versus chemotherapy alone in patients with permanently unresectable colorectal liver metastases (TransMet): Results from a multicentre, open-label, prospective, randomised controlled trial. Lancet.

[24] Bonney G.K., Chew C.A., Lodge P., Hubbard J., Halazun K.J., Trunecka P., Muiesan P., Mirza D.F., Isaac J., Laing R.W. (2021). Liver transplantation for non-resectable colorectal liver metastases: The International Hepato-Pancreato-Biliary Association consensus guidelines. Lancet Gastroenterol. Hepatol..

[25] Oh D.-Y., He A.R., Bouattour M., Okusaka T., Qin S., Chen L.-T., Kitano M., Lee C.-K., Kim J.W., Chen M.-H. (2024). Durvalumab or placebo plus gemcitabine and cisplatin in participants with advanced biliary tract cancer (TOPAZ-1): Updated overall survival from a randomised phase 3 study. Lancet Gastroenterol. Hepatol..

[26] Kelley R.K., Ueno M., Yoo C., Finn R.S., Furuse J., Ren Z., Yau T., Klümpen H.-J., Ozaka M., Verslype C. (2023). Pembrolizumab in combination with gemcitabine and cisplatin compared with gemcitabine and cisplatin alone for patients with advanced biliary tract cancer (KEYNOTE-966): A randomised, double-blind, placebo-controlled, phase 3 trial. Lancet.

[27] Valle J., Wasan H., Palmer D.H., Cunningham D., Anthoney A., Maraveyas A., Madhusudan S., Iveson T., Hughes S., Pereira S.P. (2010). Cisplatin plus Gemcitabine versus Gemcitabine for Biliary Tract Cancer. N. Engl. J. Med..

[28] Okusaka T., Nakachi K., Fukutomi A., Mizuno N., Ohkawa S., Funakoshi A., Nagino M., Kondo S., Nagaoka S., Funai J. (2010). Gemcitabine alone or in combination with cisplatin in patients with biliary tract cancer: A comparative multicentre study in Japan. Br. J. Cancer.

[29] Zhu A.X., Macarulla T., Javle M.M., Kelley R.K., Lubner S.J., Adeva J., Cleary J.M., Catenacci D.V.T., Borad M.J., Bridgewater J.A. (2021). Final Overall Survival Efficacy Results of Ivosidenib for Patients With Advanced Cholangiocarcinoma With *IDH1* Mutation: The Phase 3 Randomized Clinical ClarIDHy Trial. JAMA Oncol..

[30] Abou-Alfa G.K., Sahai V., Hollebecque A., Vaccaro G., Melisi D., Al-Rajabi R., Paulson A.S., Borad M.J., Gallinson D., Murphy A.G. (2020). Pemigatinib for previously treated, locally advanced or metastatic cholangiocarcinoma: A multicentre, open-label, phase 2 study. Lancet Oncol..

[31] Doebele R.C., Drilon A., Paz-Ares L., Siena S., Shaw A.T., Farago A.F., Blakely C.M., Seto T., Cho B.C., Tosi D. (2020). Entrectinib in patients with advanced or metastatic NTRK fusion-positive solid tumours: Integrated analysis of three phase 1–2 trials. Lancet Oncol..

[32] Hong D.S., Shen L., Van Tilburg C.M., Tan D.S.-W., Kummar S., Lin J.J., Doz F.P., McDermott R.S., Albert C.M., Berlin J. (2021). Long-term efficacy and safety of larotrectinib in an integrated dataset of patients with TRK fusion cancer. J. Clin. Oncol..

[33] Javle M., Borad M.J., Azad N.S., Kurzrock R., Abou-Alfa G.K., George B., Hainsworth J., Meric-Bernstam F., Swanton C., Sweeney C.J. (2021). Pertuzumab and trastuzumab for HER2-positive, metastatic biliary tract cancer (MyPathway): A multicentre, open-label, phase 2a, multiple basket study. Lancet Oncol..

[34] Ohba A., Morizane C., Kawamoto Y., Komatsu Y., Ueno M., Kobayashi S., Ikeda M., Sasaki M., Furuse J., Okano N. (2022). Trastuzumab deruxtecan (T-DXd; DS-8201) in patients (pts) with HER2-expressing unresectable or recurrent biliary tract cancer (BTC): An investigator-initiated multicenter phase 2 study (HERB trial). J. Clin. Oncol..

[35] Harding J.J., Piha-Paul S.A., Shah R.H., Cleary J.M., Quinn D.I., Brana I., Moreno V., Borad M.J., Loi S., Spanggaard I. (2022). Targeting *HER2* mutation–positive advanced biliary tract cancers with neratinib: Final results from the phase 2 SUMMIT basket trial. J. Clin. Oncol..

[36] Kim R.D., Chung V., Alese O.B., El-Rayes B.F., Li D., Al-Toubah T.E., Schell M.J., Zhou J.-M., Mahipal A., Kim B.H. (2020). A Phase 2 Multi-institutional Study of Nivolumab for Patients With Advanced Refractory Biliary Tract Cancer. JAMA Oncol..

[37] Lamarca A., Palmer D.H., Wasan H.S., Ross P.J., Ma Y.T., Arora A., Falk S., Gillmore R., Wadsley J., Patel K. (2021). Second-line FOLFOX chemotherapy versus active symptom control for advanced biliary tract cancer (ABC-06): A phase 3, open-label, randomised, controlled trial. Lancet Oncol..

[38] Vogel A., Saborowski A., Wenzel P., Wege H., Folprecht G., Kretzschmar A., Schütt P., Jacobasch L., Ziegenhagen N., Boeck S. (2024). Nanoliposomal irinotecan and fluorouracil plus leucovorin versus fluorouracil plus leucovorin in patients with cholangiocarcinoma and gallbladder carcinoma previously treated with gemcitabine-based therapies (AIO NALIRICC): A multicentre, open-label, randomised, phase 2 trial. Lancet Gastroenterol. Hepatol..

[39] Banales J.M., Marin J.J.G., Lamarca A., Rodrigues P.M., Khan S.A., Roberts L.R., Cardinale V., Carpino G., Andersen J.B., Braconi C. (2020). Cholangiocarcinoma 2020: The next horizon in mechanisms and management. Nat. Rev. Gastroenterol. Hepatol..

[40] Sharma A., Mohanti B.K., Chaudhary S.P., Sreenivas V., Sahoo R.K., Shukla N.K., Thulkar S., Pal S., Deo S.V., Pathy S. (2019). Modified gemcitabine and oxaliplatin or gemcitabine + cisplatin in unresectable gallbladder cancer: Results of a phase III randomised controlled trial. Eur. J. Cancer.

[41] Marabelle A., Le D.T., Ascierto P.A., Di Giacomo A.M., De Jesus-Acosta A., Delord J.-P., Geva R., Gottfried M., Penel N., Hansen A.R. (2020). Efficacy of Pembrolizumab in Patients With Noncolorectal High Microsatellite Instability/Mismatch Repair-Deficient Cancer: Results From the Phase II KEYNOTE-158 Study. J. Clin. Oncol. Off. J. Am. Soc. Clin. Oncol..

[42] Hyung J., Kim I., Kim K.-P., Ryoo B.-Y., Jeong J.H., Kang M.J., Cheon J., Kang B.W., Ryu H., Lee J.S. (2023). Treatment With Liposomal Irinotecan Plus Fluorouracil and Leucovorin for Patients With Previously Treated Metastatic Biliary Tract Cancer: The Phase 2b NIFTY Randomized Clinical Trial. JAMA Oncol..

[43] Strosberg J.R., Caplin M.E., Kunz P.L., Ruszniewski P.B., Bodei L., Hendifar A., Mittra E., Wolin E.M., Yao J.C., E Pavel M. (2021). 177Lu-Dotatate plus long-acting octreotide versus high-dose long-acting octreotide in patients with midgut neuroendocrine tumours (NETTER-1): Final overall survival and long-term safety results from an open-label, randomised, controlled, phase 3 trial. Lancet Oncol..

[44] Singh S., Halperin D., Myrehaug S., Herrmann K., Pavel M., Kunz P.L., Chasen B., Tafuto S., Lastoria S., Capdevila J. (2024). [177Lu]Lu-DOTA-TATE plus long-acting octreotide versus high-dose long-acting octreotide for the treatment of newly diagnosed, advanced grade 2–3, well-differentiated, gastroenteropancreatic neuroendocrine tumours (NETTER-2): An open-label, randomised, phase 3 study. Lancet.

[45] Strosberg J., El-Haddad G., Wolin E., Hendifar A., Yao J., Chasen B., Mittra E., Kunz P.L., Kulke M.H., Jacene H. (2017). Phase 3 Trial of ^177^ Lu-Dotatate for Midgut Neuroendocrine Tumors. N. Engl. J. Med..

[46] Sistani G., Sutherland D.E.K., Mujoomdar A., Wiseman D.P., Khatami A., Tsvetkova E., Reid R.H., Laidley D.T. (2020). Efficacy of 177Lu-Dotatate Induction and Maintenance Therapy of Various Types of Neuroendocrine Tumors: A Phase II Registry Study. Curr. Oncol..

[47] Makary M.S., Regalado L.E., Alexander J., Sukrithan V., Konda B., Cloyd J.M. (2023). Clinical Outcomes of DEB-TACE in Hepatic Metastatic Neuroendocrine Tumors: A 5-Year Single-Institutional Experience. Acad. Radiol..

[48] Egger M.E., Armstrong E., Martin R.C.G., Scoggins C.R., Philips P., Shah M., Konda B., Dillhoff M., Pawlik T.M., Cloyd J.M. (2020). Transarterial Chemoembolization vs Radioembolization for Neuroendocrine Liver Metastases: A Multi-Institutional Analysis. J. Am. Coll. Surg..

[49] Touloupas C., Faron M., Hadoux J., Deschamps F., Roux C., Ronot M., Yevich S., Joskin J., Gelli M., Barbé R. (2021). Long Term Efficacy and Assessment of Tumor Response of Transarterial Chemoembolization in Neuroendocrine Liver Metastases: A 15-Year Monocentric Experience. Cancers.

[50] Braat A.J.A.T., Kappadath S.C., Ahmadzadehfar H., Stothers C.L., Frilling A., Deroose C.M., Flamen P., Brown D.B., Sze D.Y., Mahvash A. (2019). Radioembolization with 90Y Resin Microspheres of Neuroendocrine Liver Metastases: International Multicenter Study on Efficacy and Toxicity. Cardiovasc. Interv. Radiol..

[51] Mohan H., Nicholson P., Winter D.C., O’shea D., O’toole D., Geoghegan J., Maguire D., Hoti E., Traynor O., Cantwell C.P. (2015). Radiofrequency Ablation for Neuroendocrine Liver Metastases: A Systematic Review. J. Vasc. Interv. Radiol..

[52] Caplin M.E., Pavel M., Ćwikła J.B., Phan A.T., Raderer M., Sedláčková E., Cadiot G., Wolin E.M., Capdevila J., Wall L. (2016). Anti-tumour effects of lanreotide for pancreatic and intestinal neuroendocrine tumours: The CLARINET open-label extension study. Endocr. Relat. Cancer.

[53] Kunz P.L., Graham N.T., Catalano P.J., Nimeiri H.S., Fisher G.A., Longacre T.A., Suarez C.J., Martin B.A., Yao J.C., Kulke M.H. (2023). Randomized Study of Temozolomide or Temozolomide and Capecitabine in Patients With Advanced Pancreatic Neuroendocrine Tumors (ECOG-ACRIN E2211). J. Clin. Oncol..

[54] Yao J.C., Fazio N., Singh S., Buzzoni R., Carnaghi C., Wolin E., Tomasek J., Raderer M., Lahner H., Voi M. (2016). Everolimus for the treatment of advanced, non-functional neuroendocrine tumours of the lung or gastrointestinal tract (RADIANT-4): A randomised, placebo-controlled, phase 3 study. Lancet.

[55] Chan J.A., Geyer S., Zemla T., Knopp M.V., Behr S., Pulsipher S., Ou F.-S., Dueck A.C., Acoba J., Shergill A. (2025). Phase 3 Trial of Cabozantinib to Treat Advanced Neuroendocrine Tumors. N. Engl. J. Med..

[56] Rinke A., Müller H.-H., Schade-Brittinger C., Klose K.-J., Barth P., Wied M., Mayer C., Aminossadati B., Pape U.-F., Bläker M. (2009). Placebo-Controlled, Double-Blind, Prospective, Randomized Study on the Effect of Octreotide LAR in the Control of Tumor Growth in Patients With Metastatic Neuroendocrine Midgut Tumors: A Report From the PROMID Study Group. J. Clin. Oncol..

[57] Capdevila J., Fazio N., Lopez C., Teulé A., Valle J.W., Tafuto S., Custodio A., Reed N., Raderer M., Grande E. (2021). Lenvatinib in Patients With Advanced Grade 1/2 Pancreatic and Gastrointestinal Neuroendocrine Tumors: Results of the Phase II TALENT Trial (GETNE1509). J. Clin. Oncol..

[58] Caplin M.E., Pavel M., Ćwikła J.B., Phan A.T., Raderer M., Sedláčková E., Cadiot G., Wolin E.M., Capdevila J., Wall L. (2014). Lanreotide in Metastatic Enteropancreatic Neuroendocrine Tumors. N. Engl. J. Med..

[59] Gujarathi R., Tobias J., Abou Azar S., Keutgen X.M., Liao C.Y. (2024). Peptide Receptor Radionuclide Therapy versus Capecitabine/Temozolomide for the Treatment of Metastatic Pancreatic Neuroendocrine Tumors. Cancers.

[60] NCCN Guidelines Version 2.2024 Neuroendocrine and Adrenal Tumors. February 2024. https://www.nccn.org/professionals/physician_gls/pdf/neuroendocrine.pdf.

[61] Strosberg J., Mizuno N., Doi T., Grande E., Delord J.-P., Shapira-Frommer R., Bergsland E.K., Shah M.H., Fakih M., Takahashi S. (2020). Efficacy and Safety of Pembrolizumab in Previously Treated Advanced Neuroendocrine Tumors: Results From the Phase II KEYNOTE-158 Study. Clin. Cancer Res..

[62] Janjigian Y.Y., Kawazoe A., Bai Y., Xu J., Lonardi S., Metges J.P., Yanez P., Wyrwicz L.S., Shen L., Ostapenko Y. (2023). Pembrolizumab plus trastuzumab and chemotherapy for HER2-positive gastric or gastro-oesophageal junction adenocarcinoma: Interim analyses from the phase 3 KEYNOTE-811 randomised placebo-controlled trial. Lancet.

[63] Bang Y.-J., Van Cutsem E., Feyereislova A., Chung H.C., Shen L., Sawaki A., Lordick F., Ohtsu A., Omuro Y., Satoh T. (2010). Trastuzumab in combination with chemotherapy versus chemotherapy alone for treatment of HER2-positive advanced gastric or gastro-oesophageal junction cancer (ToGA): A phase 3, open-label, randomised controlled trial. Lancet.

[64] Shitara K., Lordick F., Bang Y.J., Enzinger P., Ilson D., Shah M.A., Van Cutsem E., Xu R.-H., Aprile G., Xu J. (2023). Zolbetuximab plus mFOLFOX6 in patients with CLDN18.2-positive, HER2-negative, untreated, locally advanced unresectable or metastatic gastric or gastro-oesophageal junction adenocarcinoma (SPOTLIGHT): A multicentre, randomised, double-blind, phase 3 trial. Lancet.

[65] Shah M.A., Kennedy E.B., Alarcon-Rozas A.E., Alcindor T., Bartley A.N., Malowany A.B., Bhadkamkar N.A., Deighton D.C., Janjigian Y., Karippot A. (2023). Immunotherapy and Targeted Therapy for Advanced Gastroesophageal Cancer: ASCO Guideline. J. Clin. Oncol..

[66] Safran H.P., Winter K., Ilson D.H., Wigle D., DiPetrillo T., Haddock M.G., Hong T.S., Leichman L.P., Rajdev L., Resnick M. (2022). Trastuzumab with trimodality treatment for oesophageal adenocarcinoma with HER2 overexpression (NRG Oncology/RTOG 1010): A multicentre, randomised, phase 3 trial. Lancet Oncol..

[67] Hofheinz R.-D., Merx K., Haag G.M., Springfeld C., Ettrich T., Borchert K., Kretzschmar A., Teschendorf C., Siegler G., Ebert M.P. (2022). FLOT Versus FLOT/Trastuzumab/Pertuzumab Perioperative Therapy of Human Epidermal Growth Factor Receptor 2–Positive Resectable Esophagogastric Adenocarcinoma: A Randomized Phase II Trial of the AIO EGA Study Group. J. Clin. Oncol..

[68] Wagner A.D., Grabsch H.I., Mauer M., Romario U.F., Kang Y.-K., Bouche O., Lorenzen S., Moehler M.H., Thuss-Patience P.C., Elme A. (2023). Integration of trastuzumab (T), with or without pertuzumab (P), into perioperative chemotherapy (CT) of HER-2 positive gastric (GC) and esophagogastric junction cancer (EGJC): First results of the EORTC 1203 INNOVATION study, in collaboration with the Korean Cancer Study Group, and the Dutch Upper GI Cancer group. J. Clin. Oncol..

